# Supplementary material for: Switching on and off the spin polarization of the conduction band in antiferromagnetic bilayer transistors
Source: Nat Nanotechnol. 2025 Mar 11;20(5):609–16. doi: 10.1038/s41565-025-01872-w (PMC12095049; doi:10.1038/s41565-025-01872-w)
Supplement: Supplementary file 1 — Supplementary information. [file 41565_2025_1872_MOESM1_ESM.pdf]

nature nanotechnology

Supplementary information https://doi.org/10.1038/s41565-025-01872-w

Switching on and off the spin polarization of the conduction band in antiferromagnetic bilayer transistors

In the format provided by the authors and unedited

Table of Contents

Supplementary Fig.1-7 …………………………………………………………………..… Page 2-8
Supplementary Note 1-2 ……………………………………..……………………..… Page 9-11

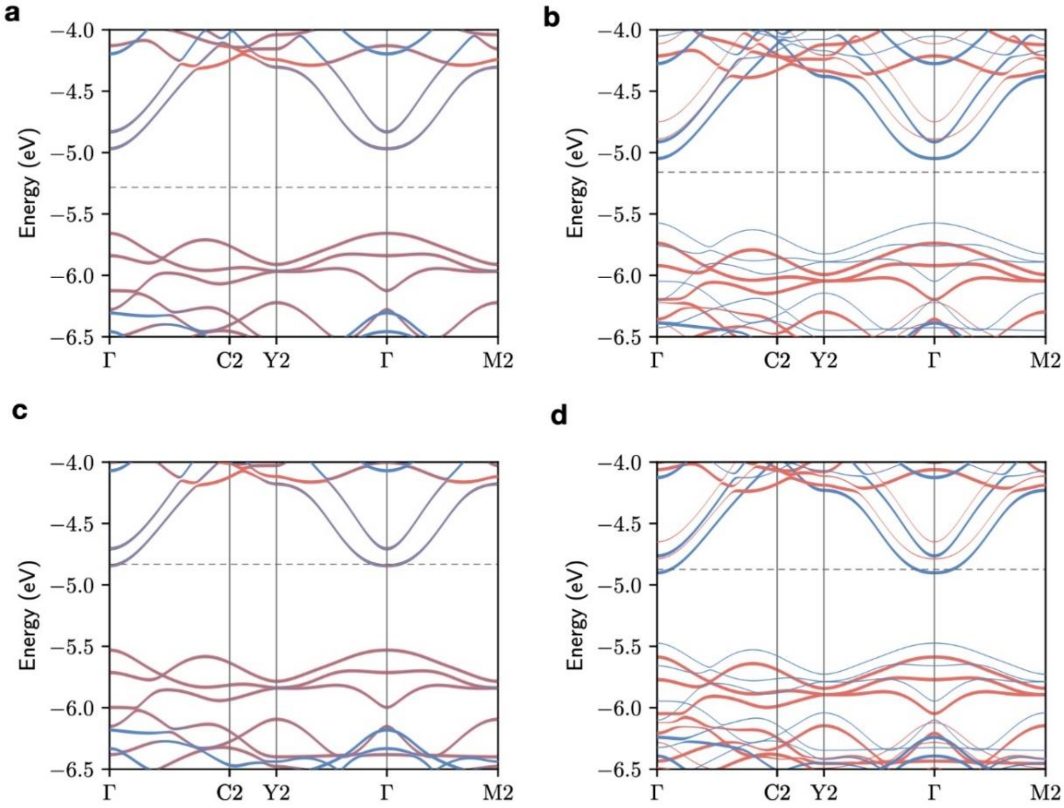

Supplementary Fig. 1. Evolution of the calculated band structure of 2 L $CrPS_4$ with doping and electric displacement field. a, Energy bands of pristine (no doping, no electric displacement field) bilayer $CrPS_4$ in the antiferromagnetic state along a high-symmetry path in the Brillouin zone. **b**, Same as in **a** but with a finite electric displacement field but no doping. **c**, Same as in **a** but in the presence of finite doping in symmetric double-gate setup without net electric displacement field. **d**, Same as in **a** but in the presence of both finite doping and finite electric displacement field. In all panels red and blue colors represent spin-up and spin-down states, while the line thickness indicates whether the corresponding electronic states are localized on the top (thick lines) or bottom (thin lines) layer. The horizontal dashed line highlights the position of the Fermi energy. The plots fully substantiate with ab-initio calculations the considerations made in the main text, illustrated by Fig. 1a, about the effect of a vertical displacement field on the energy of bands of bilayer $CrPS_4$ and about which bands get populated when doping with electrons.

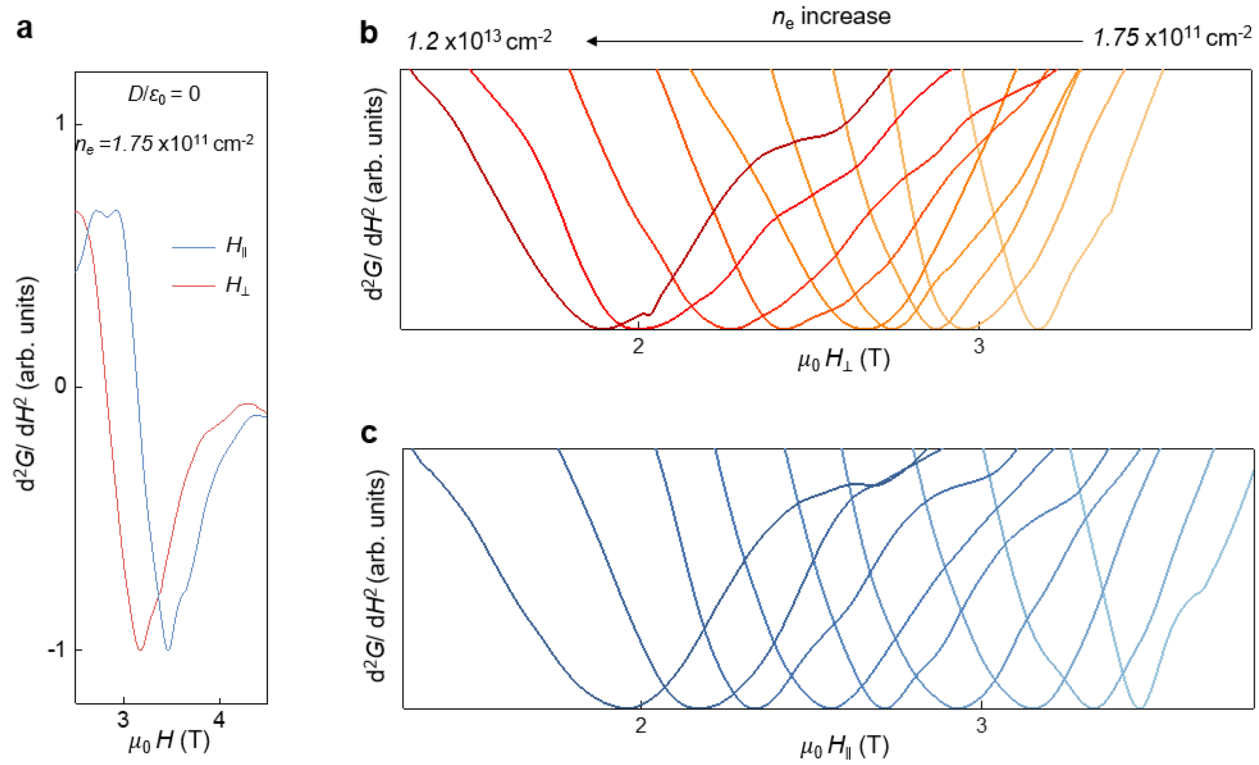

Supplementary Fig. 2. Evolution of the spin-flip field as a function of doping density at D/ε_0 = 0 V/nm. a, Second derivative of G with respect to magnetic field applied perpendicular ($H_\perp$; red curve) or parallel ($H_\parallel$, blue curve) to the plane, at $n_e = 1.75 \times 10^{11}$ cm^{-2}. The position of the minimum provides a precise determination of the spin-flip field H_{flip} (measurements done at $T = 2$K; data normalized to the minimum value for ease of comparison). **b, c,** second derivative of the conductance G with respect to magnetic field applied perpendicular ($H_\perp$, **b**) and parallel ($H_\parallel$, **c**) to the $CrPS_4$ layers at different doping density (from right to left: 1.75×10^{11} to 1.2×10^{13} cm^{-2}). The data show that the spin-flip field decreases with increasing the density of electrons accumulated in the transistor channel.

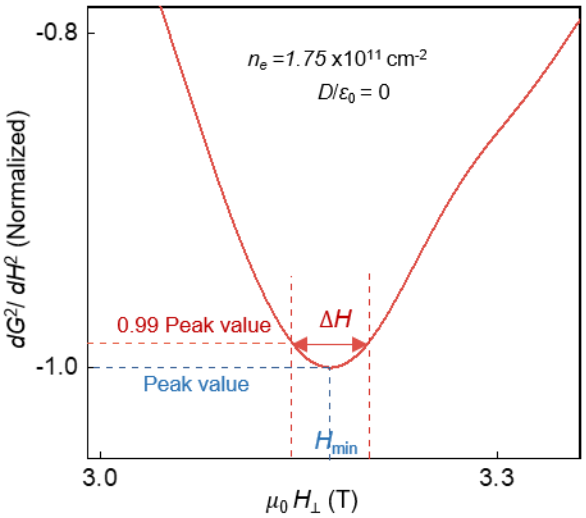

Supplementary Fig. 3. Example of error bar determination for the spin-flip field. As we explain in the main text, the value of H_{flip} is determined from the position of the minimum in the d^2G/dH^2 curve (other quantities, e.g., H_{flop}, are determined in conceptually similar ways, and the procedure outlined here to estimate the error on the value of H_{flip} applies to those cases as well). The error bar is estimated from the width of the minimum in d^2G/dH^2, ΔH, taken with a 1% criterion. Specifically, ΔH is given by the differences of the values of H on the right and left side of the minimum, for which d^2G/dH^2 deviates by 1% from the value of d^2G/dH^2 at the minimum, as illustrated in the figure. The values and error bar plotted in the figures of the main text corresponds to $H_{min} \mp 0.5\ \Delta H$.

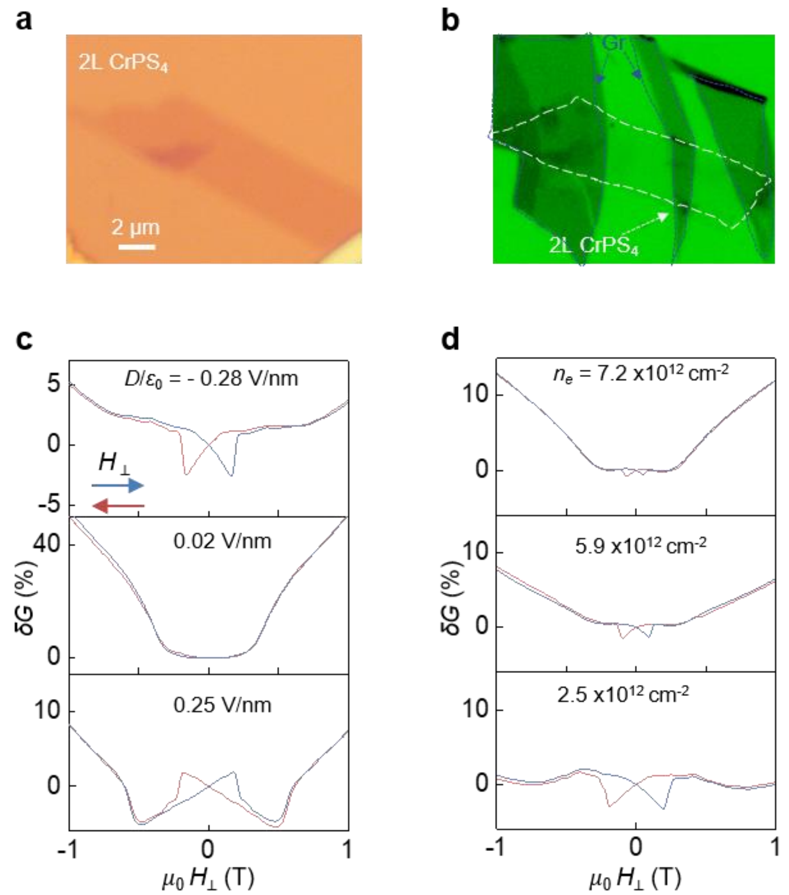

Supplementary Fig. 4. Reproducibility of the magnetotransport properties of double gate 2L $CrPS_4$ FETs. To illustrate the reproducibility of the results discussed in the main text, here we show selected data measured on a second bilayer device. **a,** Optical microscope image of an exfoliated 2L $CrPS_4$ crystal on top of a 285 nm SiO_2/Si substrate, and **b** of the assembled h-BN/ $CrPS_4$ /graphite (Gr)/h-BN heterostructure. The device structure is analogous to that of the device shown in Extended Data Fig. 1, on which the data discussed in the main text have been measured. **c**, low-field magnetoconductance δG measured at $T = 2$ K, at constant electron density (3×10^{12} cm^{-2}) for different displacement fields (-0.28 V/nm; top, 0.02 V/nm; middle and 0.25 V/nm; bottom). The behaviour of the magnetoconductance and of its hysteresis is virtually identical to the behaviour exhibited by the device discussed in the main text. **d**, δG (T= 2K) measured at constant displacement field (-0.3 V/nm) for different electron density (7.2×10^{12} cm^{-2}; top, 5.9×10^{12} cm^{-2}; middle and 2.5×10^{12} cm^{-2}; bottom). Also in this case, the evolution of the magnetoconductance and of the hysteresis reproduces what we discussed in the main text.

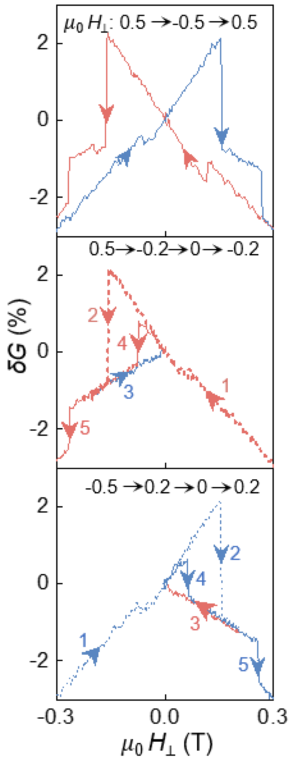

Supplementary Fig. 5. Evidence for domain formation when switching between state A and B in bilayer CrPS$_4$. Switching between states A and B depends on the precise evolution of the applied magnetic field, as it is typical for magnetization switching in ferromagnetic conductors mediated by domain formation[1]. To substantiate this statement, here we show the result of so-called minority loops measurements, in which the sweeping direction of the magnetic field is reversed before terminating the complete hysteresis loop. The resulting behavior is shown in the middle and bottom panels, in which the magnetic field is varied following the sequence 1-2-3-4-5 indicated in the figure (D/ε_0 = 0.62 V/nm; n_e = 1.5 × 10^{12} cm^{-2}; the corresponding field ranges are indicated in the figure; blue (red) corresponds to sweep up (down) of the perpendicular magnetic field). The data show that details of hysteresis are different for the minority loops as compared to the full hysteresis loop.

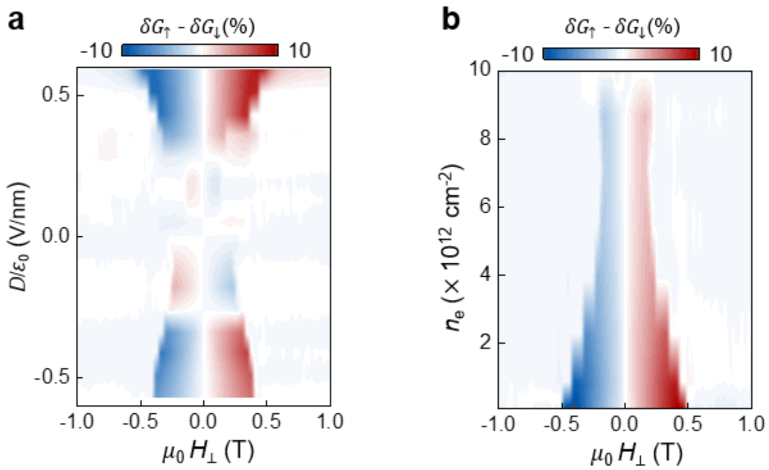

Supplementary Fig. 6. Detailed behavior of the magnetoconductance hysteresis in 4L $CrPS_4$. a, color plot of magnetoconductance hysteresis ($\delta G_{\uparrow}$ - $\delta G_{\downarrow}$) as a function of displacement field at a constant n_e =2 × 10^{13} cm^{-2}. Increasing the displacement field from -0.6 to 0.6V/nm causes the sign of the magnetoconductance hysteresis in 4L to change multiple times. This is likely because the electronic wavefunctions evolves from being distributed equally over all layers at $D/\varepsilon_0 = 0$ to be localized only the outer layer (top or bottom depending on the sign of the displacement field), causing multiple changes in the electronic magnetization. A second 4L device that we investigated exhibited the same evolution of the magnetoconductance hysteresis, with multiple changes of sign when the displacement field was varied from large negative to large positive values. **b,** color plot of magnetoconductance hysteresis ($\delta G_{\uparrow}$ - $\delta G_{\downarrow}$) measured at a constant positive displacement field ($D/\varepsilon_0 = 0.33$ V/nm) upon varying n_e (from 0.1 -10 × 10^{13} cm^{-2}). The amplitude of the magnetoconductance hysteresis decreases with n_e, similarly to the trend observed for 2L $CrPS_4$ (see Fig. 5 in main text and Supplementary Fig. 7). At the largest value of n_e reached in the experiments, a small but finite amplitude is still visible.

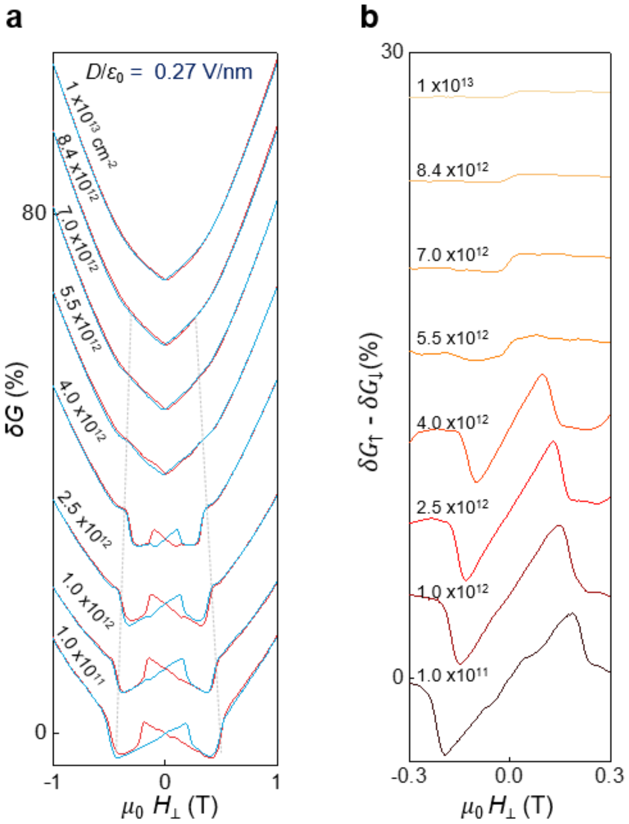

Supplementary Fig. 7. Evolution of the low-field magnetoconductance hysteresis of 2L $CrPS_4$ as a function of doping density for positive displacement field. a, low-field magnetoconductance $\delta G(H_\perp, 2\text{K})$ measured at a constant positive displacement field (D/ε_0 = 0.27 V/nm) and varying n_e (see legends in the panel). The kink associated to the spin-flop transition shifts to lower magnetic fields (indicted by the grey dashed line), becomes less pronounced, and eventually vanishes. **b**, The amplitude of the magnetoconductance hysteresis ($\delta G_\uparrow - \delta G_\downarrow$, where the arrows indicate sweep up and sweep down) decreases with n_e and eventually vanishes at $n_e \approx 8 \times 10^{12}$ cm^{-2}, in complete agreement with the behaviour observed for negative displacement fields (see Fig. 4 in main text)

Supplementary Note 1: Evolution of the energy bands of bilayer $CrPS_4$ under doping and electric field within density-functional-theory simulations

To support the scenario illustrated in the main text, including in particular Fig. 1a and the corresponding discussion in the introduction, we have performed first-principles density-functional-theory simulations of bilayer CrPS4, under different conditions achievable in a double-gate field-effect setup. A summary of the results is reported in Supplementary Fig. 1, where we show the energy bands of bilayer $CrPS_4$ in its antiferromagnetic ground state along a high-symmetry path in the Brillouin zone. Red and blue colors represent spin-up and spin-down states, while the line thickness indicates whether the corresponding electronic states are localized on the top (thick lines) or bottom (thin lines) layer.

In the pristine case without doping and without electric displacement field (panel **a**), each energy appears twice, with different color and thickness, implying that each band is two-fold spin degenerate, and that the two degenerate states with opposite spin are localized on the two different layers. Such bilayer band structure can be interpreted as two copies of the monolayer energy bands where the role of spin-up and spin-down states is exchanged in the two layers because of the opposite magnetization direction. The dashed line marks the position of the Fermi energy, that in a pristine bilayer lies in the middle of the band gap.

When an electric displacement field is added (panel **b**), the degeneracy between the two layers is lifted because of the different electrostatic potential felt by the two layers. This results in essentially a rigid shift of the energy bands of one layer (thick lines) with respect to the ones of the other layer (thin lines), and these energy bands are spin polarized. Since the system is still semiconducting, the Fermi energy continues to lie inside the gap.

Panels **c** and **d** illustrate the effect of doping on top of the situations reported in panels **a** and **b**, respectively. This means that in panel **c** doping is added without inducing a net electric displacement field, that is in a symmetric double-gate set up. While in panel **d** an asymmetric configuration is considered, where the electric displacement field across the bilayer is the same as in panel **b**.

When a pristine bilayer (**a**) is doped (**c**), the accumulated electrons are equally shared between the two degenerate bands, thus giving rise to an equal amount of doping charge on the two layers. Since the degenerate states have opposite spins, the doping carriers have no net spin polarization and the magnetization is unaffected.

On the contrary, when doping is added (**d**) on top of a situation with finite electric displacement field (**b**), the carriers first populate the lowest conduction band, which is fully spin and layer polarized. For sufficiently small doping or sufficiently large electric displacement fields, only this lower band gets occupied and there is thus 100% spin (and layer) polarization. This is the situation considered in panel **d**, where the dashed line indicates the position of the Fermi energy, showing that the accumulated electrons occupy only the lowest conduction band with a given spin projection, and are thus fully spin polarized.

Supplementary Note 2: Spin-flip and spin-flop fields within the antiferromagnetic two-site model

To describe the magnetic energy of the bilayer, in the main text we adopt the following antiferromagnetic two-site model

$$E = J\boldsymbol{M}_1\cdot\boldsymbol{M}_2 / M_s^2 - K/2\,(M_{1z}/M_s)^2 - K/2\,(M_{2z}/M_s)^2 - \mu_0\,\boldsymbol{H}\cdot(\boldsymbol{M}_1+\boldsymbol{M}_2),$$

where J is the antiferromagnetic interlayer exchange coupling, K is the magnetic anisotropy energy favoring out-of-plane orientation, $\boldsymbol{M}_1$ and $\boldsymbol{M}_2$ are the magnetization vectors of the two layers, and $\boldsymbol{H}$ is the applied magnetic field. The quantity $M_s = 2g\mu_B S$ is the saturation magnetization (per unit cell) for a single layer, which can be easily computed from the nominal valence state of Cr atoms in $CrPS_4$ (corresponding to $S = 3/2$). Upon doping we expect J and K to vary, showing a non-negligible density dependence (see Fig. 2g and Fig. 2h of the main text). In principle, also the saturation magnetization of the layers changes (in particular in the layer where the charge is predominantly accumulated). The effect is nonethless neglible for the evaluation of the spin-flip and spin-flop field, and will thus be discarded. We mention that the small but finite magnetization change associated with the doping carriers is instead crucial to set the relative energy between the otherwise degenerate states that give rise to the observed hysteresis at very low fields (shown in Fig. 3 of the main text).

Within this model the energy of the collinear AFM configuration is $E_{AFM} = -J \pm K$, where the upper (lower) sign corresponds to spins in the in-plane (out-of-plane) direction. Similarly, for the FM configuration we find $E_{FM} = J \pm K$, so that $E_{FM} - E_{AFM} = 2J$, a relation which is used in the main text to extract the interlayer exchange parameter from first-principles calculations of the total energy in the FM and AFM configurations.

In the presence of an external field applied orthogonal to the layers, the lowest energy state changes from the collinear AFM state at zero field to a spin-flop state above a spin-flop field H_{flop} given by $\mu_0 M_s H_{flop} = \sqrt{(2J-K)K}$. In such state the magnetization vectors in the two layers form the same angle θ with the vertical direction of the field but have opposite in-plane components. Minimization of the energy shows that this angle is given by

$$\theta = \arccos\left(\frac{\mu_0 M_s H}{2J-K}\right).$$

The angle decreases with increasing field, up to the spin-flip field $H_{flip}^{\perp}$ at which the two magnetization vectors lie in the direction of the field, with

$$\mu_0 M_s H_{flip}^{\perp} = 2J - K.$$

When the magnetic field is applied parallel to the layers, the spins in the two layers, from the collinear AFM state, start immediately to cant in the direction of the field, with which they form an angle α given by

$$\alpha = \arccos\left(\frac{\mu_0 M_s H}{2J+K}\right).$$

With increasing parallel field the angle α decreases, until the spins are aligned with field at the parallel spin-flip $H_{\text{flip}}^{\parallel}$, with

$$\mu_0 M_s H_{\text{flip}}^{\parallel} = 2J + K.$$

From the knowledge of the spin-flip field both in the parallel and orthogonal configurations it is then possible to extract the interlayer exchange and anisotropy energy:

$$J = \mu_0 M_s \left(H_{\text{flip}}^{\parallel} + H_{\text{flip}}^{\perp}\right)/4$$

$$K = \mu_0 M_s \left(H_{\text{flip}}^{\parallel} - H_{\text{flip}}^{\perp}\right)/2$$

which are the relations used in the main text to obtain these parameters from the measured values of the spin-flip fields.
